# Selection of neuropsychological tasks from a language test battery that optimally related to the function of each cortical area: Toward making a cognitive cortical map

**DOI:** 10.1016/j.nicl.2019.101799

**Published:** 2019-03-28

**Authors:** Yasuhiro Suzuki

**Affiliations:** Department of Neurology, Shizuoka Saiseikai general hospital, 1-1-1 Oshika, Suruga-ku, Shizuoka 422-8527, Japan

**Keywords:** Voxel-based lesion-symptom mapping (VLSM), Standard Language Test of Aphasia (SLTA), Logistic regression analysis

## Abstract

We developed a cortical language map from performance data on a language test battery in patients with brain lesions. The research problem was how to select the subtest that was most related to the function of each cortical area from the battery. When studied by voxel-based lesion-symptom mapping (VLSM), patients were divided into two groups: those with and without a lesion at each particular region. We considered the task that optimally discriminated between the two groups to be the task most related to the function of a given region. One hundred and fifty left-lesioned patients were examined using the Japanese Standard Language Test of Aphasia (SLTA), which is composed of 26 subtests. Using logistic discriminant analysis, we selected the subtest that optimally discriminated the lesioned and non-lesioned groups for each cortical region. Patients with left middle frontal gyrus (area 46) lesions were optimally discriminated from patients without lesions in that area by the speech sound–kana letter choice matching subtest. Patients with lesions in the inferior postcentral gyrus were optimally distinguished by the disturbance of word repetition. Patients with lesions in the anterior cingulate gyrus were characterized by impaired performance on the category fluency subtest. Voxel-based discriminant analysis can thus select the subtest that can be regarded as most related to the function of each cortical area.

## Highlights

• From a cognitive test battery, we selected the subtest most related to the function of each cortical area.
• We conducted voxel-based lesion-symptom mapping using t-test and logistic regression.
• The t-statistic and G-statistic can be used as indicators of differences in task performance.
• This method is useful for clarifying the primary function of each region of cortex.

## 1 Introduction

Research on higher brain function has enabled the discovery of many roles of cortical areas in cognitive functions, and more than one cognitive function is sometimes associated with a particular cortical region. There has been significant discussion as to which function is primary for a region, and which ones are secondary. Our focus in this paper was on developing a method of identifying functions with the strongest relationship to cortical areas. In functional images such as functional magnetic resonance imaging (fMRI), the signal intensity elicited by a task can be an indicator of task-region association, allowing for comparison of the association strength. The present research problem was to determine which of the candidate cognitive tasks was most associated with function in a given cortical region in the study of patients with lesions.

Voxel-based lesion-symptom mapping (VLSM, Bates et al., 2003) has been recently developed to determine local brain function using neuropsychological tests, and presents a powerful approach to this problem. VLSM was proposed as a method for judging significant statistical differences in cognitive performance between patients with and without a lesion in a specific voxel. Under this method, the task with the greatest difference in performance between patients with and without lesions is considered to be the task that is most related to a local function of the voxel.

As the indicator of difference between the two groups, the t-statistic for task performance can be considered a possible indicator of the relatedness of a region to a task. Although the t-statistic itself is not an indicator of effect size, if the sample sizes of two groups are consistent, the performance data with the highest t-statistic indicates the task with the largest effect size.

Another measure is a treatment as a classification problem into two groups. If patients are classified into groups with and without lesions by their task performance, the more accurate the discrimination is, the more closely the difference in performance is reflective of local brain function. Such discrimination can be carried out via discriminant analysis. Logistic discrimination is currently one of the most common method of discriminant analysis. The explanatory variable in the optimal fitting of the regression model to the data is considered the best discriminating variable, and goodness-of-fit is measured by the log-likelihood or G-statistic (Hosmer and Lemeshow, 2000).

In this study, we used patient performance data on a language test battery that included multiple subtests. We selected the best discriminating subtest for each cortical area using the described analysis methods, and determined the most relevant subtest pertaining to the function of each cortical area.

## 2 Method

### 2.1 Participants

We analyzed data from 150 patients (68 women) at Shizuoka Saiseikai General Hospital between 2003 and 2018. Participants were left-hemisphere stroke patients who initially presented with aphasia, and met the following criteria: speak native Japanese, have normal or corrected-to-normal vision and hearing, have at least six years of education, and have no major psychiatric or neurological disorders. Exclusion criteria were also applied based on behavioral performance or neuroimaging findings; these criteria are described later in the text (2.2, 2.3). Finally, 150 patients were selected. One hundred and forty-eight of the patients were right-handed; there were 116 cases of ischemic stroke, and 34 cases were hemorrhagic. Further demographic data are provided in Table 1. Informed consent was obtained from all participants, and this study was reviewed and approved by the Shizuoka Saiseikai General Hospital Ethics Committee.

**Table 1:** Demographic patient data.

Variable	Min.	Q1	Median	Mean	Q3	Max.
Age (years)	17	63	70	69.3	78	94
Lesion size (ml)	3	21	43	46.4	59	197
Imaging days post onset	0	0	1	3.8	3	97
SLTA examination days post onset	2	7	11	15.2	18	76

SLTA: Standard Language Test of Aphasia.

### 2.2 Behavioral measures

**Fig. 1:**
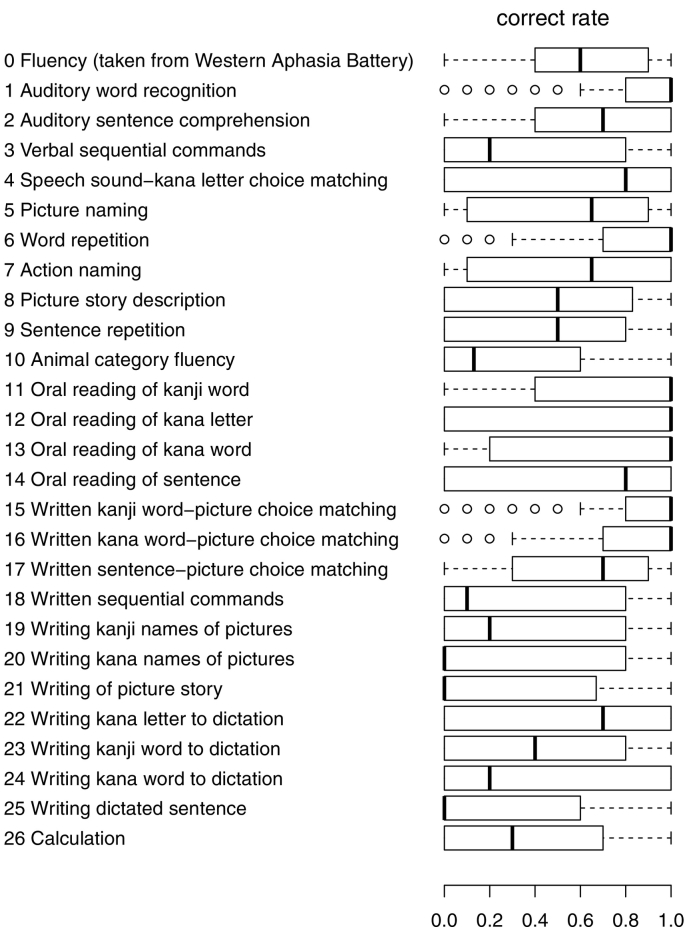
Patients' correct rates for each subtest in the Standard Language Test of Aphasia (SLTA) depicted as a boxplot.

Patients were evaluated using the Standard Language Test of Aphasia (SLTA; Japan society for higher brain dysfunction, 1997). The SLTA is a comprehensive Japanese language test battery that includes 26 subtests for hearing (subtests 1–4), speaking (subtests 5–14), reading (subtests 15–18), writing (subtests 19–25), and calculation (subtest 26) abilities. The SLTA subtests and the distribution of the correct rates among participants in this study are shown in Fig. 1; a summary of the test manual is shown in the Appendix. Because the SLTA lacks a spontaneous speech fluency measure, speech fluency was assessed by the fluency item from the Western Aphasia Battery (WAB, Kertesz, 1982), which was numbered as subtest 0 in this study. Consequently, 27 items (26 SLTA subtests and the WAB fluency subtest) were examined.

Cases were excluded if all SLTA subtests were performed perfectly, which indicated that the patient was not aphasic. Severely aphasic patients who could not perform any of the subtests in the SLTA were also excluded. Such cases were excluded because this study intended to compare the discrimination power between subtests. The performance data for the above cases were regarded meaningless for this purpose, and we considered that these data cause the information bias resulting from ceiling or floor effects.

### 2.3 Neuroimaging

All lesions were confirmed with CT (*n* = 33) or 1.5 Tesla MRI (*n* = 117; DWI 106, FLAIR 5, T2*WI 4, T2WI 2). Cases were limited to those with small lesion volumes below 200 ml, because VLSM deliberately eliminates effects by lesion except the voxel of interest (Bates et al., 2003). Thus, the smaller the lesion is, the smaller the influence of other irrelevant areas is, and the resulting area more accurately reflects the true functional area.

Brain magnetic resonance angiography (MRA) was also evaluated in all cases imaged by MRI. Cases with more than 90% stenosis or obstruction of the truncal arteries (the carotid artery or trunk of the middle cerebral artery) were excluded because their entire middle cerebral artery region could be symptomatically ischemic and cause neuropsychological disorders, even if visual ischemic lesions were not recognized on MRI. Cases with simultaneous lenticulostriate artery lesions were also excluded because their truncal artery was likely to be obstructed transiently, even if truncal arteries were patent on MRA in the neuroimaging period (Bladin and Berkovic, 1984).

### 2.4 Lesion reconstruction and regions of interest (ROI)

**Fig. 2:**
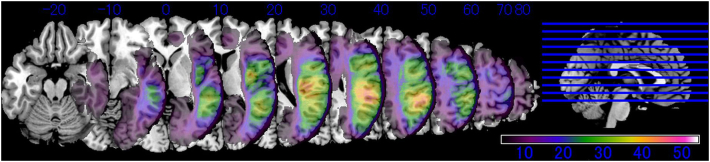
Lesion overlay maps for all patients included in the study. This figure shows only the voxels for which a minimum of five individuals had lesions. The color bar represents the number of subjects. The site of maximum overlap is in the supramarginal gyrus, where 53 patients had damage. The site of maximum overlap in the medial cortical surface was in the medial frontal cortex, where 18 patients had damage.

All lesions were mapped using MRIcron software (Rorden et al., 2007; http://people.cas.sc.edu/rorden/mricron, last accessed 1 December 2018) and were drawn manually by a single researcher, on slices of normalized T1-weighted template MRI scans from the Montreal Neurological Institute (MNI), distributed with the MRIcron toolset. The researcher was blind to the participant's cognitive performance. The MNI coordinates of lesions were visually determined on the template; Fig. 2 shows the number of overlapping lesions. We excluded voxels for which fewer than five patients had lesions from the following analyses.

We limited the region of interest (ROI) to the cortex because the existing software for VLSM does not implement discrimination analysis. The ROI was set to a depth of 1 cm from the cortical surface along the X-axis of the MNI coordinates because the number of lesioned patients was largest, as shown in Fig. 2, and the statistical power was highest at that depth. This study consequently used two-dimensional *pixel*-based lesion-symptom mapping, instead of typical three-dimensional voxel-based lesion symptom mapping. For the MNI Y-Z coordinates, the examined pixel pitch was preset to five-millimeter increments. This study differs on these points from the typical VLSM method which uses neuroimaging voxels. We investigated a total of 813 pixels.

### 2.5 Statistical analysis and selection of optimal discriminating subtests for each pixel

Before the statistical analyses, we controlled for the severity of aphasia in each patient. However, the Aphasia Quotient is not defined in the SLTA as in the Western Aphasia Battery. We therefore regarded the average correct rate of all subtests for the patient as the indicator of aphasia severity. The average correct rate was subtracted from the raw correct rate for each subtest, and this value was defined as the adjusted correct rate, which was used in the following analyses.

Two methods were employed to select the subtest with optimal discrimination between patients with and without a lesion at each pixel. The first method was Welch's *t*-test for subtest performance between two groups. Of the 27 subtests, we selected the subtest that fulfilled the following two conditions: (1) patients with a lesion performed poorer than those without a lesion; and (2) the subtest had the highest t-statistic among the tests.

The second method was logistic discrimination analysis for two groups. The explanatory variables (independent variables) for the logistic regression analyses were the previously described adjusted correct rates for the 27 subtests. The response variables (dependent variables) were whether or not the pixel was lesioned (0 = intact, 1 = lesioned). We were able to obtain 27 candidate univariate logistic models, including the explanatory variables as performance on the 27 subtests for each pixel. We selected final models that fulfilled the following two conditions: (1) presence of a lesion reduced performance on the subtest; and (2) the model optimally discriminated between the two groups. This was determined by maximizing the log-likelihood or G-statistic, which is defined as −2 × (the log-likelihood of the constant-only model minus log-likelihood of the candidate model) (Hosmer and Lemeshow, 2000). The G-statistic follows the chi-square distribution with degrees of freedom that are equal to the difference in the number of explanatory variables between the two models. The likelihood ratio chi-square test can then be conducted using the G-statistic.

Statistical cut-off thresholds were determined with the alpha set to 0.05. One thousand data permutations were used to correct the significant cut-offs and in order to control family-wise error (FWE) for multiple comparisons across the whole brain (Holmes et al., 1996; Kimberg et al., 2007). There is no known VLSM software that can compare the statistical values described above, so all analyses were performed using R statistical software, version 3.40 (http://www.r-project.org/, last accessed 1 December 2018). An R script written by Aoki Shigenobu (http://aoki2.si.gunma-u.ac.jp/R/all.logistic.html, last accessed 1 December 2018) was modified and used to select the logistic model with the highest G-statistic from the candidate models.

## 3 Results

### 3.1 VLSM maps for individual subtests

**Fig. 3:**
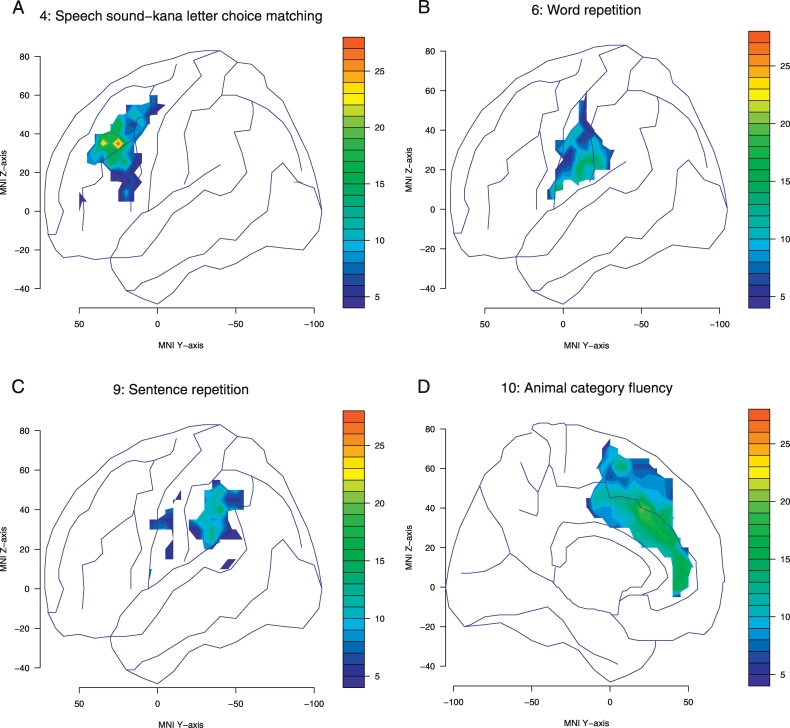
G-statistic maps for four subtests. The areas shown in color exceeded the critical threshold for significance (no correction). Maps are superimposed on the MNI space. The colored bars indicate G values. (A) Speech sound–kana letter choice matching. The region with the highest G values was the middle frontal gyrus. (B) Word repetition. The region with the highest G values was the inferior postcentral gyrus. (C) Sentence repetition. The region with the highest G values was the supramarginal gyrus. (D) Category fluency. The region with the highest G values was the anterior cingulate gyrus.

We made 27 VLSM maps for the 27 examined tasks (not all maps are included in the present study). Fig. 3 shows the VLSM maps for representative performance on four subtests (4, 6, 9, and 10), calculated using the G-statistic. The subtest performance was associated with lesions in the middle frontal gyrus, inferior postcentral gyrus, supramarginal gyrus, and anterior cingulate gyrus, respectively. Similar figures were obtained by plotting the *t*-statistic distribution (data not shown). The effect size of the logistic regression could be shown as a correlation coefficient or odds ratio. These resembled the G-statistic maps, and were omitted.

In the next section, we describe the tasks that had the greatest discrimination power at each pixel. We used the highest statistical values to create a map by overlaying the maps of individual subtest performance.

### 3.2 Selection of subtests with the greatest differences in performance at each pixel

**Fig. 4:**
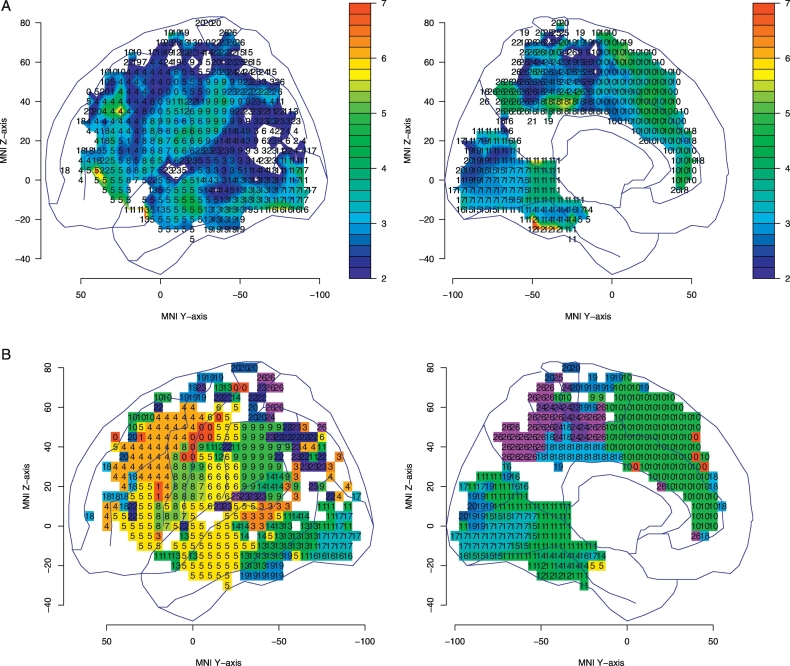
T-statistic map of subtest with the highest t-statistic for each pixel. Each pixel number indicates the subtest number that optimally discriminated between the lesioned and non-lesioned groups. (A) T-statistics are shown using color. The areas shown in color exceeded the critical threshold for significance (no correction). The color bars indicate the t value. For multiple comparisons, the t-statistic exceeded the threshold (*t* = 3.68) in the middle frontal gyrus, inferior parietal cortex, and anterior cingulate gyrus. (B) Pixel colors are matched to subtest numbers in the area above the significant threshold. Refer to Fig. 1 for the meaning of the subtest numbers.

To select the subtest with the greatest difference in performance between patients with and without lesions at each pixel, we used two statistics (*t* and G). Fig. 4A shows the map of the STLA subtest numbers with the highest t-statistic for performance between patients with and without lesions for each pixel; the t-statistic is shown by color. The threshold of the *t*-statistic was 1.98 if multiple comparisons were not considered. Fig. 4B was created by matching the pixel color to each subtest number in the significant region. The permutation threshold of the t-statistic to control for FWE across the whole cortex was 3.68; which was exceeded in the middle frontal gyrus, anterior inferior parietal lobe, and anterior cingulate gyrus, etc.

**Fig. 5:**
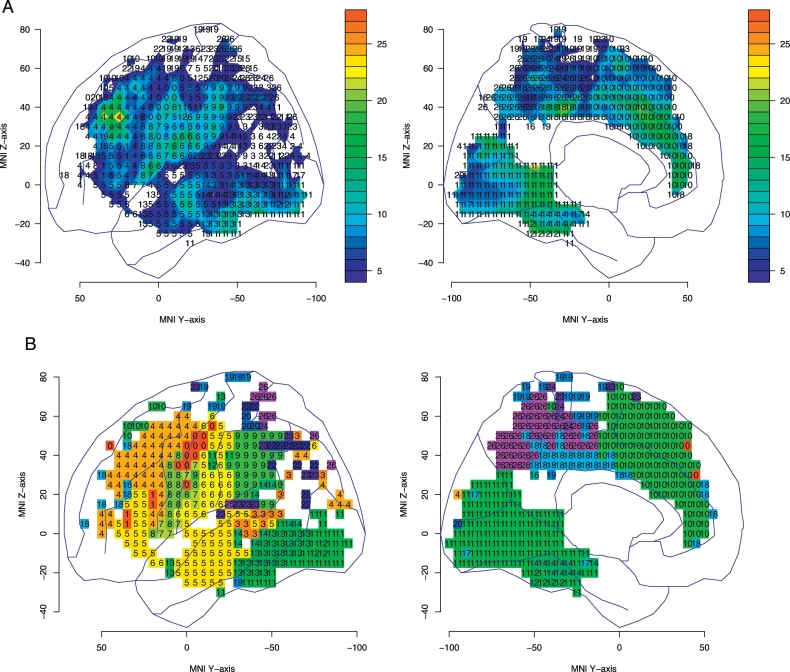
G-statistic map of subtest with the highest G-statistic for each pixel. Each pixel number indicates the subtest number that optimally discriminated between the lesioned and non-lesioned groups. (A) G-statistic are shown using color. The areas shown in color exceeded the critical threshold for significance (no correction). The color bars indicate the G value. For multiple comparisons, the G-statistic exceeded the threshold (G = 11.1) in the middle frontal gyrus, inferior parietal cortex, and anterior cingulate gyrus. (B) Pixel colors are matched to subtest numbers in the area above the significant threshold. Refer to Fig. 1 for the meaning of the subtest numbers.

Fig. 5A shows a map of the SLTA subtest numbers that optimally discriminated between patients with and without lesions at each pixel in the logistic models; the G-statistic is shown by color. The G-statistic follows a chi-square distribution, and a G-statistic over 3.84 means that the 95% CI of the odds ratio does not include 1, if multiple comparisons are not considered. Fig. 5B was made by matching the pixel color of each subtest number in the significant region. The permutation threshold of the G-statistic to control for FWE across the whole cortex was 11.1. The area above the threshold of G-statistic was similar to the area above the threshold of t-statistic. When sex, age, and lesion volume were also included as explanatory variables in the logistic model, age was the most discriminating variable at 26 pixels, and sex was the most discriminating variable at a single pixel. However, the discriminative power of these variables was not very strong, and the G-statistics were below eight. Lesion volume was not the most discriminating variable at any pixel.

As the shared region shown by both analyses, damage to the middle frontal gyrus (around area 46) is best discriminated from an absence of damage in this region by performance on subtest 4, speech sound–kana letter choice matching. In the inferior postcentral gyrus (the slice of z coordinate 15–30 in the MNI space), word repetition (subtest 6) was the most discriminating subtest; meanwhile sentence repetition (subtest 9) disturbance was most characteristic of patients with supramarginal lesions. Category fluency (subtest 10) was the most discriminating subtest in the anterior cingulate gyrus region.

## 4 Discussion

### 4.1 General discussion

This study selected subtests for which performance was most distinct between patients with and without a lesion at each pixel using two analysis methods. We considered the selected subtests to be reflective of function at each pixel. Similar subtests were selected at many pixels using the t-statistic and G-statistic. Prior to this study, we were interested in the difference of statistical power between these measures. The areas of significance in both analyses were similar, in other words, these analyses had comparable statistical power. The *t*-statistic is easily calculated; meanwhile, logistic regression has the advantage that odds ratios and predicted probability can be calculated, and it can also be applied to multivariate analysis. Each analysis has its place based on its characteristics.

Although the selected subtests do not directly represent the function of the pixels examined, they were selected as having the strongest correlation with function among the candidate subtests. Though we selected the subtests that we thought would be optimal for discriminating patients from SLTA, further selection of subtests would be possible from other language batteries or other non-language cognitive batteries. The accumulation of data from analogous studies may contribute to the determination of local brain function.

The symptom evaluation period has influenced results in analogous studies. *Re*-organization occurs after stroke, and language performance often changes in the early stage after a stroke event. This study was performed during the acute stage of stroke (2–76 days), and thus was not likely to include patients who had recovered entirely. Thus, to some extent, our data reflect the status of individuals before re-organization was complete. However, in the present study, we did not precisely control the examination date. We would like to acknowledge this limitation.

It is desirable that the results of lesion studies are supported by neuroimaging data such as fMRI, which was not conducted in the study. Although the results of both methods are generally the same, inconsistencies do occur. One reason for this is that the activated regions in fMRI data do not always correspond to the regions that are necessary to perform the task (Rorden and Karnath, 2004). For example, Silva et al. (2018) reviewed the validity of fMRI prior to neurosurgical cortical resection, and reported that fMRI could not be completely substituted for more invasive tests such as electrocortical stimulation or the Wada test. However, the presence of cognitive impairments in patients with lesions represents a direct demonstration that the affected regions are required for function. This is an advantage of lesion studies compared with fMRI studies.

### 4.2 Specific discussion

In terms of specific cortical areas, lesions in the middle frontal gyrus were shown to be associated with impairments on subtest 4, “speech sound–kana letter choice matching”. To our knowledge, the present study is the first to show an association between the frontal cortex and a sound-letter matching task. Subtest 4 is partially similar to subtest 12, “oral reading of kana letter” and subtest 22, “writing kana letter to dictation.” However, subtest 4 is unique in that its input information modalities take two forms: speech sounds and letters. This subtest can be regarded as representative of the integration function of auditory sound and visual letter information. Recently, the inferior frontal gyrus has been shown to be involved in the audiovisual integration of semantic information (Plakke and Romanski, 2014). The present study suggested that audiovisual integration of meaningless symbols, such as sound-letter matching, requires function of the middle frontal gyrus rather than that of the inferior frontal gyrus.

Patients with lesions in the inferior postcentral gyrus were characterized by word repetition disturbance, and those with supramarginal gyrus lesions were characterized by impairments in sentence repetition. Fig. 3 shows VLSM with logistic regression for each subtest separately. The area found to be significantly relevant to word repetition was also primarily located around the inferior postcentral gyrus. Previous VLSM studies have shown a relationship between repetition and the posterior perisylvian region, the supramarginal gyrus and superior temporal gyrus regions in particular (Fridriksson et al., 2010; Baldo et al., 2012; Dell et al., 2013; Rogalsky et al., 2015; Wilson et al., 2015; Pilkington et al., 2017). The postcentral gyrus has not previously been considered to be very important.

As a possible reason for this discrepancy, we initially took differences in analytical methods into consideration. That is, this study employed logistic regression of the correct rate adjusted by the mean correct rate of all subtests. However, VLSM of the raw correct rates using *t*-tests also showed that region around the postcentral gyrus had significant relevance to word repetition, similar to what is shown in Fig. 3 (*t*-statistic maps for this measure are omitted).

Therefore, the discrepancies between the present findings and those of previous studies may be attributed to differences in participants, rather than analytical methods. Our participants had relatively small and acute lesions; severely damaged patients with no response on the SLTA were excluded. This difference in participant exclusion criteria may have caused the difference in results. Our results are similar to that of Kümmerer et al. (2013), which showed repetition disturbance primarily in postcentral lesion patients. Their study was similar to ours in that participants were in the acute stage and had relatively small lesions. These factors appear to be involved in the difference in results found in our study compared with some other studies.

Meanwhile, fMRI studies (Price, 2012) do not favorably correspond with these lesion studies. For example, Buchsbaum et al. (2011) showed that the Sylvian-parietal-temporal region (SPt) was the only site that was both lesioned in conduction aphasia patients and activated during a phonological short-term memory task in controls. This discrepancy may reflect, in part, the finding that increased blood flow does not necessarily indicate that the region is necessary to perform the task (Rorden and Karnath, 2004).

Additionally, we found different brain areas related to word repetition and sentence repetition in the present study. This may be explained by the finding that short-term memory is more heavily involved in sentence repetition (McCarthy and Warrington, 1990; Majerus, 2013) than in word repetition, and that speech articulation is more strongly involved in word repetition (Markiewicz and Bohland, 2016; Leonard et al., 2016) than in sentence repetition.

Finally, the anterior cingulate region showed the strongest relationship with subtest 10, the category fluency task. Previous VLSM studies have also shown an association between this area and category fluency (Kinkingnéhun et al., 2007; Cristofori et al., 2015; Geisseler et al., 2015). Wagner et al. (2014) also demonstrated the relationship between word fluency and activation in the anterior cingulate gyrus using functional MRI. Patients with such lesions have been known to present with mutism and transcortical motor aphasia (Kumral et al., 2002). This study compared statistics between subtest performance, and showed that the category fluency task most typically represented the aphasic feature for anterior cingulate lesions in the SLTA. Although several other cortical areas were shown to be related to some of the other tasks, discussion of these relationships has been omitted.

### 4.3 Limitations

One limitation of this study was that the description of results was limited to the cortex, and presented only as pixels at every MNI 5 mm pitch. This means this study is not VLSM in the usual sense. The primary reason for this is that VLSM software capable of comparing statistics from 27 tasks does not exist, and secondly there are difficulties in displaying three-dimensional results. Thirdly there are limitations in voxel-based lesion-symptom mapping itself. When calculating statistical values at specific voxels in VLSM, the effect of other lesions is eliminated (Bates et al., 2003). Therefore, when patient lesions were very large and they included a significant lesion that is unrelated to the ROI, patient performance may not directly reflect the function of the ROI (Mah et al., 2014). If VLSM study includes data for patients whose lesions are so large that the lenticular striate artery region are affected, it could cause confusion in interpreting the association between the cortical lesion and the symptoms. Such patients were therefore deliberately excluded from this study, and the region examined was limited to the cortex. Moreover, we did not include a normal control in the present study, and every participant had lesions elsewhere, either inside or outside of the ROI. VLSM itself is limited in that it neglects the possible impact of deficits from other cortical areas and resulting influence on subtest performance.

Our assessment of behavioral performance represents another limitation. The nature of deficits can impact performance in unique ways (error types), and can also yield important information regarding cortical functioning. However, we did not examine these factors in the present study. Another limitation is that the detailed data regarding years of education were not available in this study, although we can be sure that all participants completed at least 6 years.

## 5 Conclusions

In conclusion, this study identified the optimally discriminating subtests in a language test battery for estimating the primary function of cortical areas based on differences between patients with and without lesions in each cortical region. In addition to the conventional perspective of identifying the region responsible for a function, the approach of identifying the primary function of a region, as in this study, will contribute to furthering brain function research.

## Appendix A Appendix

Summary of Standard Language Test of Aphasia (SLTA).

The SLTA consists of 26 subtests shown below. In this study, we used 27 items including WAB fluency as subtest 0.

1: Auditory word recognition. Pointing to a correct card from ten picture cards based on the name presented verbally by the examiner. List of pictures: egg, horse, car, sun, socks, telephone, water, glass, cap, and house.

2: Auditory sentence comprehension. Pointing to a correct card from ten picture cards presenting acting subjects based on the sentence presented verbally by the examiner. For example, “A student gets a certificate from the teacher.”

3: Verbal sequential commands. Token test using ten common objects: handkerchief, mirror, comb, pencil, scissors, toothbrush, 100-yen coin, key, match, and pen.

4: Speech sound–kana letter choice matching. Pointing to a correct card from ten kana letter cards according to examiner's speech sound: “me,” “a,” “ho,” “ta,” “ya,” “nu,” “ki,” “ne,” “se,” and “re.”

5: Picture naming. Naming of 20 picture cards: book, pencil, dog, clock, rice, top, mountain, newspaper, airplane, goldfish, drug, drum, desk, crocodile, lantern, *torii* (gateway to a Shinto shrine), bamboo, deer, *fusuma* (papered sliding door in a Japanese house), and *kadomatsu* (New Year's pine decoration).

6: Word repetition. Repeating the word after the examiner (the same list used in subtest 1).

7: Action naming. Naming the action in ten picture cards: sleeping, reading, drinking, swimming, riding, flying, writing, striking, crossing, and inflating.

8: Picture story description. The participant was asked to tell a story about a four-panel comic strip using four key words, ‘walk’, ‘hat’, ‘fly’, ‘pick up’, or similar words. Full score: perfect sentences including the four key words. Four-fifths score: incomplete or paraphasic sentences including the four key words. Three-fifths score: sentences with three key words. Two-fifths score: sentences with two key words. A fifth score: only one word.

9: Sentence repetition. Repeating a sentence formed with two to six words after the examiner.

10: Animal category fluency. Listing 15 animal names verbally in a minute. Paraphasic words were approved as correct if the examiner could understand the meaning of the word.

11: Oral reading of kanji word. Oral reading of five kanji-word cards: dog, book, clock, newspaper, pencil.

12: Oral reading of kana letter. Oral reading of ten kana-letter cards (the same cards used in subtest 4).

13: Oral reading of kana word. Oral reading of five kana-word cards (the same list used in subtest 11).

14: Oral reading of sentence. Oral reading of sentence formed with two or three words, selected from the sentences used in subtest 2.

15: Written kanji word–picture choice matching. Reading a kanji word card and pointing to a correct picture from ten picture cards (the same cards used in subtest 1).

16: Written kana word–picture choice matching. Reading a kana word card and pointing to a correct picture from ten picture cards (the same cards used in subtest 1).

17: Written sentence–picture choice matching. Reading a sentence card and pointing to a correct picture from ten picture cards (the same cards used in subtest 2).

18: Written sequential commands. Reading a sentence card and performing token test using 10 common objects (the same instructions used in subtest 3).

19: Writing kanji names of pictures. Writing kanji names of five picture cards (the same lists used in subtest 11).

20: Writing kana names of pictures. Writing kana names of five picture cards (the same cards used in subtest 19).

21: Writing of a picture story, using the same comic picture used in subtest 8.

22: Writing kana letter to dictation (the same list used in subtest 4).

23: Writing kanji word to dictation (the same list used in subtest 11).

24: Writing kana word to dictation (the same list used in subtest 11).

25: Writing dictated sentences (two or three phrase sentences, contents are used in subtest 8).

26: Calculation. Reading a calculation problem card and calculating the answer on paper. Oral answers were also approved. Operations include addition, subtraction, multiplication, and division.

In the speech subtest (5–7, 9, and 11–14), although mere dysarthria did not warrant a demerit point, a point was deducted for a mora error associated with apraxia of speech. In the writing subtest (19–25), writing with either hand was approved if one hand was paralyzed.

## Appendix B Supplementary data

Performance and lesion data of 150 patients Image 1
